# Determinants of pregnant women's compliance with alcohol guidelines: a prospective cohort study

**DOI:** 10.1186/1471-2458-12-777

**Published:** 2012-09-13

**Authors:** Amy E Anderson, Alexis J Hure, Jennifer R Powers, Frances J Kay-Lambkin, Deborah J Loxton

**Affiliations:** 1 Priority Research Centre for Gender, Health and Ageing, University of Newcastle, University Drive, Callaghan, NSW, 2308, Australia; 2 Priority Research Centre for Translational Neuroscience and Mental Health Research, University of Newcastle, University Drive, Callaghan, NSW, 2308, Australia; 3 National Drug and Alcohol Research Centre, University of New South Wales, Randwick, NSW, 2032, Australia

**Keywords:** Alcohol drinking, Guidelines, Health behaviour, Patient compliance, Pregnancy, Prenatal care, Prevalence, Women's health

## Abstract

**Background:**

In 2009, Australian alcohol guidelines for pregnancy changed from low to no alcohol intake. Previous research found a high proportion of pregnant Australian women drank during pregnancy; however, there has been limited investigation of whether pregnant women comply with 2009 alcohol guidelines. The purpose of this study was to provide an assessment of pregnant women’s compliance with 2009 Australian alcohol guidelines and identify predictors of such compliance, including previous drinking behaviour.

**Methods:**

Cross-sectional analysis of prospective data from the 1973–1978 cohort of the Australian Longitudinal Study on Women’s Health was conducted. Women aged 30–36 years who were pregnant at the 2009 survey and had data on alcohol use were included (n = 837). Compliance with 2009 alcohol guidelines for pregnancy was defined as no alcohol intake. Predictors of compliance were analysed using multivariate logistic regression, controlling for area of residence, in three separate models to account for multicollinearity between measures of previous alcohol intake (compliance with 2001 guidelines; frequency and quantity; bingeing). Private health insurance, household income, and illicit drug use were entered into all models and retained if significant.

**Results:**

72% of pregnant women did not comply with the 2009 alcohol guidelines and 82% of these women drank less than seven drinks per week, with no more than one or two drinks per drinking day. The odds of complying with abstinence increased by a factor of 3.48 (95% CI 2.39-5.05) for women who previously complied with the 2001 alcohol guidelines and decreased by a factor of 0.19 (95% CI 0.08-0.66) if household incomes were $36,400 or more. In other models the odds of complying were lower for women who consumed alcohol before pregnancy at least weekly (OR = 0.40, 95% CI 0.25-0.63) or binged (OR ≥ 0.18, 95% CI 0.10-0.31) and were higher for those who abstained (OR = 45.09; 95% CI 8.63-235.49) prior to pregnancy.

**Conclusion:**

Most pregnant women did not comply with alcohol guidelines promoting abstinence. Prior alcohol behaviour was the strongest predictor of compliance during pregnancy, suggesting alcohol use should be addressed in women of child-bearing age. The study is limited by the relatively short timeframe between the official introduction of the 2009 guidelines and the date the surveys were sent out. Widespread dissemination of the guidelines may be necessary to help increase guideline compliance by pregnant women.

## Background

Public health guidelines are intervention strategies aimed at bringing about health behaviour change at a population level [1]. They synthesize the best available evidence to assist healthcare providers and individuals to make informed decisions. The constant nature of research means that public health guidelines change over time and may vary by country, depending on culture and healthcare priorities. Change over time and international discrepancy is very prominently demonstrated by the guidelines on drinking alcohol during pregnancy. In Australia the 1992 alcohol guidelines suggested women abstain from alcohol during pregnancy [2]. In 2001, these guidelines were revised to condone low levels of drinking [3].

The 2001 guidelines contained the following recommendations for pregnant women, or those that may soon become pregnant:

• may consider not drinking at all;

• most importantly should never become intoxicated;

• if they choose to drink, over a week, should have less than 7 standard drinks, AND, on any one day, no more than 2 standard drinks (spread over at least two hours);

• should note that the risk is highest in the earlier stages of pregnancy, including the times from conception to the first missed period [3].

In February 2009, the Australian guidelines were again changed to state that “not drinking is the safest option” [4]. A draft of these 2009 guidelines was made available for public consultation back in 2007 and was advertised in major newspapers, through media coverage, and on the National Health and Medical Research Council’s website [4]. Australia’s current guideline on abstinence during pregnancy is similar to those in the US, Canada and Denmark [5-7], but differs to the guidelines promoted in the UK [8].

Research assessing alcohol use under the previous guidelines found the vast majority (around 80%) of Australian women did consume alcohol during pregnancy [9-11]. Analysis from the Australian Longitudinal Study on Women’s Health (ALSWH) data collected from 1996 to 2006 found that drinking during pregnancy occurred regardless of a guideline change from abstinence (1992 to 2001) to low-level drinking (2001 to 2009) [10]. Similarly, Danish research found no significant change in consumption among pregnant women when guidelines changed in 1999 from abstinence to no more than one drink per day [12]. Factors such as pre-pregnancy alcohol intake, smoking during pregnancy and stage of pregnancy were found to be significant predictors of compliance with alcohol guidelines [10]. Although previous alcohol consumption has been found to be a consistent contributing factor to drinking during pregnancy [13] its measurement in studies has not been consistent. Frequency [14-17], quantity [15,17,18], and binge episodes [14,19] have all been used as measures of previous drinking behaviour. However, previous compliance to alcohol guidelines has not been independently assessed. A recent report investigated drinking behaviour of Australian women in 2010, but did not account for alcohol consumption prior to pregnancy [20]. To date, no studies have investigated whether pregnant Australian women comply with the 2009 guideline to not drink during pregnancy, accounting for previous alcohol intake.

The purpose of this project was to assess pregnant women’s compliance with 2009 Australian alcohol guidelines [4] and to identify determinants of compliance. Of particular interest, we examined whether previous guideline compliance predicted subsequent compliance to alcohol guidelines during pregnancy.

## Methods

Population-based prospective data from women born between 1973 and 1978 (the 1973–1978 cohort) from the ALSWH were analysed cross-sectionally in 2011. Ethical clearance for the ALSWH was obtained from the Universities of Newcastle and Queensland (Ethics approvals H0760795 and 2004000224). Women were originally randomly sampled, with intentional oversampling from rural areas, through the national health insurer (Medicare Australia) database in 1996 and invited to participate in a 20 year longitudinal study. The women were aged 18–23 years at the time of recruitment and were broadly representative of women of the same age in the Australian population [21,22]. The cohort completed self-report surveys in 1996, 2000, 2003, 2006, and 2009. Further details of sampling and recruitment methods have been reported elsewhere [21,22].

Cross-sectional analysis of data from survey five in 2009 was conducted for this project, with survey four (2006) data utilized to identify previous behaviour. Women were included in descriptive analyses if they had reported a pregnancy and completed alcohol items at survey five (2009). Only women with self-reported pregnancies were included in order to analyse women’s behaviour in the context of their knowledge of the pregnancy. Further analyses that included measures of previous behaviours, such as smoking and alcohol consumption, was limited to women that completed survey four (2006).

The 2009 surveys were mailed out on the 31 March 2009 and on average were returned within three months (range 0–14 months). About 58% of the original sample from the baseline survey completed the 2009 survey. At the baseline survey, women who completed the 2009 survey were more likely than non-responders to have never smoked (54% vs. 45%) and had ≥12 years education (70% vs. 65%) [23]. However, there were no differences between women who completed the 2009 survey and non-responders with regards to age, marital status, or area of residence at baseline [23]. Based on previous analyses of potential attrition bias within the ALSWH, it is highly unlikely that attrition rates would have led to any significant bias in the relationships among the variables [24].

Health-related and sociodemographic factors were investigated in relation to alcohol guideline compliance. Pre-pregnancy behaviours were only calculated for women who were not pregnant or breastfeeding at survey four. Pre-pregnancy behaviours included: frequency and quantity of alcohol use, and binge drinking status. Previous smoking status and compliance with alcohol guidelines were also assessed for women that completed survey four. Previous compliance for women who were not pregnant or breastfeeding at survey four was defined as those who had drank on average two or less drinks per day, no more than 14 drinks per week, never more than four drinks on one day, and had at least one alcohol free day a week [3]. Women who were pregnant or breastfeeding at survey four were classified as compliant with alcohol guidelines if they drank less than two drinks per day, had less than seven drinks per week, and had at least one alcohol free day per week [3]. Abstainers were included in the assessment of previous alcohol use as the national alcohol guidelines are intended for the population as a whole.

Health-related characteristics from survey five that were investigated as potential predictors of guideline compliance included: stage of pregnancy, parity, gravidity, smoking status during pregnancy, and illicit drug use. Sociodemographic variables included: highest educational attainment, marital status, employment status, household income, rurality, and private health insurance.

The quantity of alcohol use was not a primary outcome for this analysis, but it was used to describe the non-compliant sample of women. Quantity of alcohol use was measured by the item “On a day when you drink alcohol, how many standard drinks do you usually have?” (1 or 2 drinks per day, 3 or 4 drinks per day, 5 to 8 drinks per day, 9 or more drinks per day). The latter three categories were combined, and a category of “does not drink” was imputed for participants who had answered “I never drink alcohol” on the alcohol frequency item.

### Primary outcome

Pregnant women’s compliance with the 2009 Australian alcohol guidelines was the primary outcome measure. As mentioned above, the 2009 guidelines had been made available in draft form and were widely advertised in 2007 [4]. Upon their release in February 2009, the guidelines were disseminated to state and territory health departments [4]. Compliance was defined as not drinking any alcohol while pregnant. Participants were categorized as pregnant if they selected any of the following responses to the question “Are you currently pregnant?”: less than 3 months, 3 to 6 months, or more than 6 months. Alcohol consumption was measured with the frequency item “How often do you usually drink alcohol?”(I never drink, less than once a month, less than once a week, on 1 or 2 days a week, on 3 or 4 days a week, every day). Pregnant women were dichotomized, with only those answering “I never drink” classified as “compliant” with 2009 guidelines [4]; all others were non-compliant.

### Statistical analysis

Analyses were conducted using SPSS (version 19.0). For all univariate analyses, data were weighted by area of residence at survey one to account for purposeful oversampling from non-urban areas. Pearson Chi-square tests were used to examine the associations between compliance to alcohol guidelines and each sociodemographic and health-related characteristic. Factors significantly related (p < 0.05) to compliance were entered into multivariate logistic regression models using a backward stepwise approach with a cut-point of 0.05. All models were adjusted for area of residence at baseline by forcing it into the model at step one. Three models were run to account for multicollinearity between measures of previous alcohol intake. The first model (Model A) included women regardless of pregnancy or breastfeeding status at survey four. The second and third models (Models B and C) pertain only to women who were not pregnant or breastfeeding during survey four to enable measurement of pre-pregnancy factors. Women with and without missing data were compared with regards to the dependent variable, and potential bias in the dependent variable was investigated for women purposefully excluded from models B and C compared with those included in the models. These analyses of bias yielded no significant differences (results not shown).

## Results

**Figure 1:**
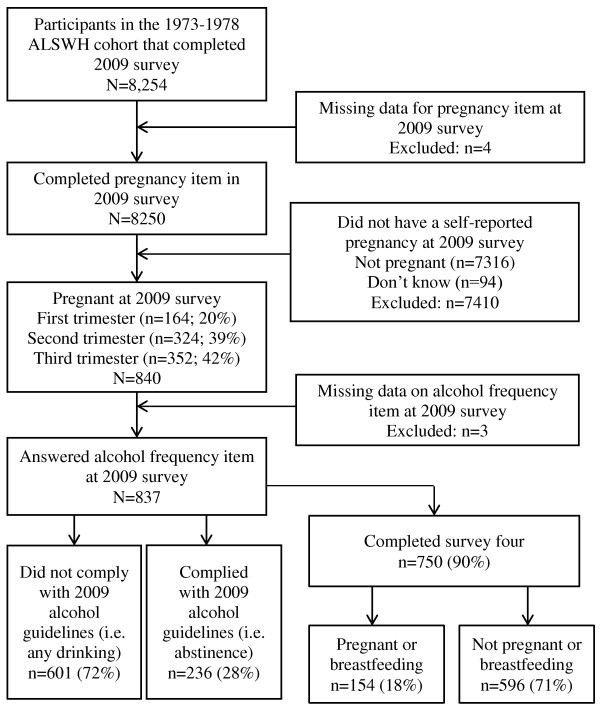
Flowchart of sample selection from the Australian Longitudinal Study on Women’s Health (ALSWH).

Figure 1 shows the selection process for the sample. The majority of pregnant women (72%; n = 601) consumed alcohol and therefore were considered non-compliant. The majority (82%; n = 491) of pregnant women that consumed alcohol had drank less than seven drinks per week, with no more than one or two drinks per drinking day.

Table 1 contains the characteristics of pregnant women categorized by compliance with alcohol guidelines. Compliant women were more likely to have lower household incomes and were slightly less likely to be privately insured. Other sociodemographic characteristics were similar between the two groups. Compliant women were more likely to have never used illicit drugs, never binged on alcohol prior to pregnancy, been non-drinkers before pregnancy, and previously complied with alcohol guidelines. Compliant women were less likely to have consumed alcohol at least once a week before pregnancy.

**Table 1:** **Sociodemographic and health-related characteristics**^**a**^**of pregnant women (N=837) by compliance with 2009 alcohol guidelines**[4]

	**Compliant**	**Non-compliant**	**Total**	**p-value**
Previous compliance with 2001 alcohol guidelines (n=736)^b^				<0.01
Non-compliant	76 (36%)	355 (68%)	431 (59%)	
Compliant	136 (64%)	169 (32%)	305 (41%)	
Frequency of pre-pregnancy alcohol use (n=589)^c^				<0.01
Less than weekly or did not drink	123 (70%)	176 (43%)	299 (51%)	
At least once a week	52 (30%)	238 (58%)	290 (49%)	
Quantity of pre-pregnancy alcohol use (n=582)^c^				<0.01
Does not drink	40 (23%)	2 (<1%)	42 (7%)	
1 to 2 drinks per drinking day	88 (51%)	268 (65%)	356 (61%)	
3 or more drinks per drinking day	44 (26%)	140 (34%)	184 (32%)	
Pre-pregnancy binge status (n=583)^c^				<0.01
Never binged or did not drink	101 (58%)	87 (21%)	188 (32%)	
Binged less than once a month	45 (26%)	185 (45%)	230 (40%)	
Binged once a month or more often	27 (16%)	138 (34%)	165 (28%)	
Education - highest qualification achieved (n=817)				0.61
Year 10 or lower	10 (4%)	19 (3%)	29 (4%)	
Year 12/trade/apprenticeship/certificate/diploma	70 (31%)	169 (29%)	239 (30%)	
University degree	149 (65%)	400 (68%)	549 (68%)	
Marital status (n=829)				0.68
Married	198 (85%)	498 (83%)	696 (84%)	
De facto	32 (14%)	90 (15%)	122 (15%)	
Never married/separated/divorced/widowed	2 (1%)	9 (2%)	11 (1%)	
Employment status (n=821)				0.25
No paid work	64 (28%)	139 (24%)	203 (25%)	
Paid work	169 (73%)	449 (76%)	618 (75%)	
Household income (n=766)				<0.01
$0 - $36,399	17 (8%)	12 (2%)	29 (4%)	
$36,400 - $77,999	44 (20%)	103 (19%)	147 (19%)	
$78,000 - $155,999	115 (53%)	288 (52%)	403 (53%)	
$156,000 or more	40 (19%)	147 (26%)	187 (24%)	
Rurality (ARIA+; n=794)				0.17
Major cities	119 (54%)	332 (61%)	451 (59%)	
Inner regional	60 (27%)	133 (25%)	193 (25%)	
Outer regional	37 (17%)	63 (12%)	100 (13%)	
Remote or very remote	6 (3%)	14 (3%)	20 (3%)	
Private health insurance (n=836)				0.03
No	62 (26%)	118 (20%)	180 (22%)	
Yes	173 (74%)	483 (80%)	656 (79%)	
Trimester (n=836)				0.18
First	37 (16%)	125 (21%)	162 (19%)	
Second	91 (39%)	232 (39%)	323 (39%)	
Third	108 (46%)	243 (41%)	351 (42%)	
Parity (number of live births; n=743)				0.42
No previous live births	67 (31%)	150 (28%)	217 (29%)	
One or more previous live births	147 (69%)	379 (72%)	526 (71%)	
First pregnancy (Gravidity; n=823)				0.88
Primigravida(First pregnancy)	55 (24%)	144 (24%)	199 (24%)	
Multigravida(Previous pregnancies)	176 (76%)	448 (76%)	624 (76%)	
Smoking status (n=836)				0.88
Non-smoker	229 (97%)	581 (97%)	810 (97%)	
Smoker	7 (3%)	19 (3%)	26 (3%)	
Previous smoking status (n=748)				0.55
Non-smoker	187 (87%)	472 (89%)	659 (88%)	
Smoker	28 (13%)	61 (11%)	89 (12%)	
Illicit drug use (n=836)				<0.01
Never used	113 (48%)	191 (32%)	304 (36%)	
Ever used	123 (52%)	409 (68%)	532 (64%)	

^a^ All variables, except rurality, were weighted by area of residence to account for oversampling from rural areas.

^b^ Compliance to 2001 NHMRC alcohol guidelines regardless of pregnancy status [3].

^c^ Only for women who were not pregnant or breastfeeding at survey 4 (N=596).

Table 2 contains the factors entered into multivariate models (Models A, B, and C) of guideline compliance. After controlling for area of residence in Model A, pregnant women were less likely to comply with alcohol guidelines if they had household incomes of $36,400 or more. The odds of complying with guidelines during pregnancy increased by a factor of 3.48 (95% CI 2.39-5.05) for women who previously complied with the 2001 alcohol guidelines.

**Table 2:** **Multivariate logistic regressions**^**a **^**of previous drinking behaviour on pregnant women’s compliance with 2009 alcohol guidelines**

**Model A (n=611 out of 750 potential participants)**^**b**^	**(Adjusted OR with 95% CI)**
Previous compliance with 2001 alcohol guidelines	
Non-compliant	Reference
Compliant	3.48 (2.39-5.05)
Household income	
$0 - $36,399	Reference
$36,400 - $77,999	0.26 (0.11-0.66)
$78,000 - $155,999	0.26 (0.11-0.62)
$156,000 or more	0.19 (0.08-0.50)
**Model B (n=479 out of 596 potential participants)**^**c**^	**(Adjusted OR with 95% CI)**
Frequency of pre-pregnancy alcohol use	
Less than weekly or did not drink	Reference
At least once a week	0.44 (0.29-0.69)
Quantity of pre-pregnancy alcohol use	
Did not drink	45.09 (8.63-235.49)
1 to 2 drinks per drinking day	Reference
3 or more drinks per drinking day	0.90 (0.57-1.42)
**Model C (n=480 out of 596 potential participants)**^**c**^	**(Adjusted OR with 95% CI)**
Pre-pregnancy binge status	
Never binge or did not drink	Reference
Binge less than once a month	0.21 (0.13-0.34)
Binge once a month or more often	0.18 (0.10-0.31)

^a^ All models were adjusted for area of residence to account for oversampling from rural areas. Illicit drug use, private health insurance, and household income were entered into all models. Illicit drug use and private health insurance were not significant in any of the models and household income was not significant in Models B and C.

^b^ All women that also completed 2006 survey regardless of pregnancy/breastfeeding status.

^c^ Only women who were not pregnant or breastfeeding at the 2006 survey.

In Model B (Table 2), only frequency and quantity of pre-pregnancy alcohol use remained in the model. Pregnant women who had consumed alcohol at least once a week before pregnancy were 56% less likely to comply with alcohol guidelines during pregnancy relative to those drinking less than weekly. Quantity of pre-pregnancy alcohol use was only significant when comparing abstainers to drinkers. Compared with women who drank 1 to 2 drinks per drinking day, women who abstained prior to pregnancy were 45 times more likely to comply with alcohol guidelines during pregnancy. There was no significant difference in compliance between the two drinking groups (1 to 2 drinks versus 3 or more drinks per drinking day).

For Model C (Table 2), pregnant women who had previously binged before pregnancy had a decreased odds of complying with alcohol guidelines. The decrease in odds was significant regardless of the frequency of the binge behaviour (monthly or less than monthly). The contributions of other factors to the model were not significant.

## Discussion

Most Australian women continue to drink during pregnancy despite a national guideline that recommends abstinence. Measures of previous alcohol use were the strongest predictors of compliance. Weekly or binge drinking and previously drinking more than recommended predicted non-compliance with guidelines during pregnancy. Women’s previous compliance with alcohol guidelines, regardless of pregnancy or breastfeeding status at that time, meant they were three and a half times more likely to comply during pregnancy. Contrary to previous research which found pre-pregnancy drinks per drinking day to be a strong predictor of consumption during pregnancy [15], this study found the predictive value of quantity of alcohol consumed on a drinking day prior to pregnancy was only applicable when comparing women who drank versus abstainers. An increased quantity of alcohol per drinking day among those who did drink was not itself predictive of guideline compliance in pregnancy. Frequency of pre-pregnancy alcohol use, however, was strongly predictive of such compliance. This supports previous research which found that the frequency, rather than the quantity, of pre-pregnancy alcohol consumption is more useful in predicting alcohol use during pregnancy [16,25]. These findings may help to simplify the assessment of women of childbearing age who may be at risk of consuming alcohol if they become pregnant by focusing on how often they drink, rather than how much they usually drink.

By using prospective data before and during pregnancy, this population-based study provided a broadly representative prevalence of pregnant women’s compliance with alcohol guidelines. This is one of the first studies to assess whether the abstinence recommendation in the 2009 guidelines has been adopted by pregnant women. It is reasonable to assume that there may be some bias in this study’s estimates as only women with a recognised pregnancy were included. Considering a larger proportion of women drink during the pre-recognition phase of pregnancy [18,20,26,27], it is likely that this exclusion criteria may have led to an overestimation of compliance. In contrast to the 72% of women reporting drinking during pregnancy in this study, a report based on the 2010 National Drug Strategy Household Survey (NDSHS) found only 28% of Australian women over 31 reported drinking after pregnancy recognition, while 57% drank during some stage of pregnancy [20]. It is possible that a proportion of the 72% of non-compliant women in our study were consuming alcohol due to a lack of awareness of the revised alcohol recommendations due to the timing of the survey. However, discrepancy between the current study and the findings from the NDSHS may be partially attributed to a difference in measurement techniques. The ALSWH obtained information at the time of pregnancy, whereas NDSHS used a retrospective recall of the drinking behaviour that occurred in pregnancies within the past 12 months [20]. The ALSWH utilised a larger sample of pregnant women (N = 837) in a more defined age group (30–36 years) compared with the sample of women in the NDSHS (n = 434) that were relatively comparable in age (31 years or over).

Prior research found that 80% of Australian women were compliant with the 2001 alcohol guidelines which condoned low alcohol intake [10], yet this study only found a 28% compliance rate with current guidelines. Given the majority (82%) of drinkers drank at low levels, a higher proportion of this study’s sample would have been classified as compliant with the 2001 alcohol guidelines. Similarly in the UK, where pregnant women are told to avoid alcohol in the first trimester and then limit alcohol to one to two drinks once or twice a week [8], only 29% of women in their first trimester complied with the recommendations of early abstinence, whereas 94% of women in later pregnancy adhered to the low alcohol intake recommendation [28]. It appears that in Australia and the UK pregnant women are far less likely to comply with recommendations for no alcohol intake. In contrast, the US and Canada have maintained strong consistent messages of alcohol abstinence for pregnant women and have found that about 89% and 86% of pregnant women, respectively, complied with alcohol guidelines [29,30]. The high proportion of Australian women that continue drinking during pregnancy suggests that there has not been a large scale uptake of the evidence-based recommendation to abstain from alcohol. Previous research supports the notion that guidelines do not necessarily impact drinking behaviour [10,12], emphasizing that the creation of guidelines alone is not sufficient in altering population behaviour.

This study confirmed findings that previous alcohol consumption is one of the best predictors of prenatal use of alcohol [10,13]. Similarly, a recent Swedish study found that higher pre-pregnancy scores on the Alcohol Use Disorders Identification Test (AUDIT) were predictive of alcohol use during pregnancy [31]. In addition to the usual forms of alcohol assessment found in the literature (i.e. frequency [14-17], quantity [15,17,18], and binge status [14,19]) this study has taken a novel approach by examining previous compliance to alcohol guidelines. By doing so, the current study was able to show a pattern of non-compliant behaviour.

### Limitations

This study is limited by the age range (30–36 years) of participants. Considering the mean age of Australian mothers is 30 years and there is a national trend of an increase in the age of mothers [32], the results are likely to be generalisable to a large proportion of pregnant Australian women. There were missing data in some analyses; however, analyses of bias yielded no significant difference in the outcome of interest due to missing or excluded cases. Self-report may have led to response bias in the under-reporting of alcohol use. However, self-report has been found to be more accurate than physicians’ medical records in identifying prenatal alcohol use [33]. Furthermore, the confidential nature in using a unique identifying code, as was done in this study, has been found to be equally effective in obtaining a high rate of self-reported alcohol use by pregnant women compared with using a purely anonymous technique [34].

This study was within the confines of a large longitudinal study which led to one of the major limitations. There was a relatively short timeframe between when the 2009 guidelines were introduced and when the surveys were sent out. However, draft guidelines were available and widely publicised as early as 2007. Previous research conducted in late 2008 to early 2009 has shown that health professionals were passing on an abstinence message to pregnant clients, consistent with the 2009 guidelines [35]. Additionally, participants on average took about three months to return their surveys, with some taking up to 14 months. Seeing as how women were asked about their alcohol use when they were pregnant, rather than asking them to recall their entire pregnancy, it is believed that this study has gathered an accurate measure of drinking during pregnancy at the time the surveys were completed, which occurred under the 2009 guidelines. Whether the guidelines were properly disseminated is a topic for further research but does not limit the fact that the 2009 guidelines were in place when the women were surveyed about their behaviour.

### Practice implications

Alcohol behaviours should be assessed before women become pregnant because pre-pregnancy alcohol use and previous compliance with guidelines predict whether Australian women will comply with guidelines during pregnancy. General practitioners (GPs) are ideally suited to assess alcohol intake in women of childbearing age. GPs are the gatekeepers to the Australian healthcare system; 19% of their clients are women of childbearing age (15–45 years) and average consultation times range from 14–15 minutes [36]. Best practice clinical guidelines suggest that pregnant women, or those who may become pregnant, should be provided with information about potential consequences of prenatal alcohol use in order to make an informed decision [8,37]. However, a random sample of Australian health professionals found that only a quarter of providers routinely provided such information [38]. Awareness and familiarity of, and attitudes towards clinical guidelines have been found to affect health professionals’ adherence to them [39].

It may be necessary for policy makers to implement strategies to effectively disseminate the alcohol guidelines for pregnant women to ensure they are both implemented by the healthcare system and adopted by the general population. Such strategies may include the use of local opinion leaders to address barriers and encourage best practice among health professionals [40]. Additionally, mass media campaigns could be developed as they have been found to be effective in other public health initiatives such as reducing alcohol-related crashes [41] and increasing initiation of and positive attitudes towards breastfeeding [42]. US authorities have suggested that in addition to mass media campaigns other universal prevention strategies, such as policy-driven warning labels on alcoholic beverages and other strategies to reduce overall consumption for the population, may be useful in helping to prevent alcohol-exposed pregnancies [43]. Studies from Scandinavian countries have reported that mass media is the number one information source regarding alcohol use in pregnancy for pregnant women [17,44]. It has also been found that pregnant women believe a health professional could best communicate this information [44] and women are comfortable discussing alcohol use with healthcare providers [35]. Currently, no mass media campaign or other universal prevention strategies exist in Australia to promote the most recent alcohol guidelines for pregnant women, stressing not only a need for public health promotion but also the importance of healthcare professionals in disseminating this public health message.

Based on the results of this study, GPs may find it useful to initiate a conversation about alcohol use by asking women about their usual alcohol consumption (e.g. when not pregnant) as a lead in to assessing their current alcohol use. If women report usually drinking more than the recommended guidelines or usually drink on a weekly basis, then the GP can use that context to provide them with information about the potential consequences of alcohol use during pregnancy and the national recommendation for abstinence. For women of childbearing age, healthcare providers could offer brief motivational interviewing which has been found to reduce the risk of alcohol exposed pregnancies [45]. GPs may consider using educational and psychological interventions for their pregnant clients, which have been found to assist pregnant women in abstaining from alcohol [46].

## Conclusion

Proper dissemination of guidelines and recommendation uptake by pregnant women are needed to ensure guideline compliance. However, more information is needed to determine why so many pregnant women are not complying with the current alcohol guidelines. It is not known whether women are aware of these guidelines and if so whether compliance is due to a purposeful adherence to the guidelines or a result of choosing to abstain for other reasons. Other countries with less conservative alcohol guidelines may wish to confirm whether a pattern of non-compliance also exists among pregnant women in their region. Additionally, dissemination, adoption, and promotion of current alcohol guidelines are most likely inadequate given the present findings. Further research is needed to understand the pathway that exists between policymakers and pregnant women to determine why there is such a low rate of compliance with alcohol guidelines.

## Abbreviations

ALSWH: Australian Longitudinal Study on Women’s Health; GP: General practitioner; NDSHS: National Drug Strategy Household Survey.

## Competing interests

The authors declare that they have no competing interests.

## Authors’ contributions

AA, DL, JP, AH and FK-L all made substantial contributions to the conception and design of the study. AA conducted the analysis under guided supervision by JP and DL. AA, DL, JP and AH made substantial contributions to the interpretation of the data. AA drafted the manuscript. All authors contributed to the revision of the manuscript. All authors read and have given approval for the final manuscript.

## Pre-publication history

The pre-publication history for this paper can be accessed here:

http://www.biomedcentral.com/1471-2458/12/777/prepub
